# Heterogeneous Stress Driven by Environmental Filtering Maintains Plant Diversity Along an Arid Riparian Gradient

**DOI:** 10.1002/ece3.73745

**Published:** 2026-06-10

**Authors:** Zidong Zhang, Zhifang Xue, Tiantian Qin, Shengtianzi Dong, Hanyue Wang

**Affiliations:** ^1^ College of Life Science Shihezi University Shihezi China; ^2^ Ministry of Education Key Laboratory of Xinjiang Phytomedicine Resource Utilization Shihezi China; ^3^ Xinjiang Production and Construction Corps Key Laboratory of Oasis Town and Mountain‐Basin System Ecology Shihezi China

**Keywords:** pH, plant functional strategy, riparian ecosystem, river continuum concept, soil phosphorus

## Abstract

Environmental stress is generally expected to suppress biodiversity, yet how heterogeneous environmental filtering regulates plant community assembly in arid riparian ecosystems remains poorly understood. Here, we investigated herbaceous plant communities and associated soil factors along riparian environmental gradients of the Ulungur River in arid northwestern China, aiming to assess whether and how severe but spatially heterogeneous stress conditions can maintain high local diversity. Non‐metric multidimensional scaling (NMDS), generalized linear models (GLM), and random forest regression were used to disentangle the relative contributions of spatial location and soil stress in shaping species turnover and diversity patterns. Our results showed that local plant alpha diversity unexpectedly peaked in downstream river segments characterized by severe soil salinization–alkalization and phosphorus limitation (pH up to 8.88, total phosphorus as low as 0.70 g/kg), challenging the widespread negative stress‐diversity relationship. NMDS ordination revealed pronounced species turnover along the riparian gradient, with the greatest spatial dispersion observed in upstream plots (mean Bray–Curtis dissimilarity = 0.569) due to high habitat heterogeneity. GLM further revealed that elevation was the strongest common negative factor for all diversity metrics (standardized coefficients *β* < −0.5, all *p* < 0.001), whereas soil organic carbon (SOC) and cation exchange capacity (CEC) had independent positive effects on halophyte richness. The random forest results corroborated the GLM findings and suggested that pH and total phosphorus influence diversity through non‐linear or interactive effects. Functional group analyses demonstrated that downstream communities were predominantly assembled from a distinct pool of stress‐tolerant halophytic species. Together, these findings suggest that multidimensional and spatially heterogeneous stress landscapes can maintain high local plant diversity by selectively filtering species with specific tolerance strategies while enhancing spatial species turnover. Our study highlights the critical role of environmental filtering in sustaining biodiversity under severe conditions and provides new insights into community assembly processes in arid riparian ecosystems.

## Graphical abstract

Environmental stress is generally expected to reduce biodiversity, yet its effects under spatially heterogeneous conditions remain unclear. Along an arid riparian gradient of the Ulungur River, we show that local herbaceous plant diversity peaks in downstream reaches despite severe salinization–alkalization and phosphorus limitation, driven by spatially structured environmental filtering that enhances species turnover and supports coexistence of stress‐tolerant taxa.

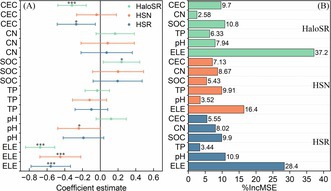

## 1 Introduction

Riparian ecosystems are critical ecological corridors that sustain biodiversity and ecosystem functioning in arid regions, and they are highly sensitive to environmental stress and disturbance (Riis et al. 2020; Berdugo et al. 2022). However, whether intense stress inevitably results in biodiversity loss across all riparian contexts remains an open and actively debated question. Environmental stress is a central driver of community assembly and has been incorporated into several influential ecological frameworks, including the Intermediate Disturbance Hypothesis (Fox 1979) and the River Continuum Concept (RCC; Vannote et al. 1980). These theories emphasize gradual environmental change along river systems and predict peak biodiversity under moderate disturbance or resource availability, typically in mid‐reach segments (Lite et al. 2005; Richardson 2019). Increasing evidence, however, suggests that under severe environmental conditions, stress does not always reduce diversity and may instead alter species coexistence mechanisms (Gross et al. 2024). Trait‐based theories, such as Grime's CSR framework, provide a potential mechanistic basis for these patterns by proposing reorganization toward stress‐tolerant strategies under severe stress (Grime 1974). Despite these advances, empirical evidence explaining non‐linear reorganization in community assembly along arid river continua remains limited (Souahi et al. 2022). Importantly, these reorganizations are likely driven not only by the intensity of environmental stress but also by its spatial heterogeneity, which can modify how environmental filtering operates along river systems.

In arid regions, soil phosphorus (P) frequently constrains plant growth more strongly than nitrogen, contrasting with nutrient limitation patterns observed in humid ecosystems (Elser et al. 2007; Kumar et al. 2019). At the same time, intense evaporation and salt accumulation resulting from natural weathering and anthropogenic inputs often lead to elevated soil pH and salinity, particularly in downstream river segments (Liu et al. 2023). These processes generate interacting soil stressors, whereby soil salinization–alkalization not only directly affects plant physiology (Yang et al. 2022; J. Wang et al. 2022) but also indirectly limits phosphorus availability (Jiang et al. 2023). Although the individual effects of phosphorus limitation and salinity on plant communities are well documented (Ma et al. 2012; Ceulemans et al. 2017), their combined influence as a multidimensional soil stress landscape defined by salinization–alkalization and phosphorus limitation along arid river systems remains poorly quantified. Such interacting soil stressors are expected to generate spatially heterogeneous filtering regimes, potentially leading to non‐linear responses in local plant diversity along river continua.

To address these gaps, we conducted a basin‐wide survey of herbaceous plant communities and soil properties along the Ulungur River in arid northwestern China. Although species composition has been reported for portions of this basin (Zhang et al. 2025), how environmental filtering operates along the full river continuum and how interacting soil stressors jointly influence plant diversity remain unclear. We tested the hypothesis that spatially heterogeneous and interacting soil stressors modify environmental filtering regimes, thereby generating non‐linear changes in community assembly that allow stress‐tolerant species to coexist and sustain high local diversity under severe conditions. Specifically, we aimed to (1) quantify changes in community composition and species turnover along the river continuum, (2) examine whether downstream communities are characterized by distinct stress‐tolerant assembly patterns, and (3) disentangle the direct and indirect effects of riparian position and key soil factors on plant diversity.

## 2 Materials and Methods

### 2.1 Study Area

**FIGURE 1:**
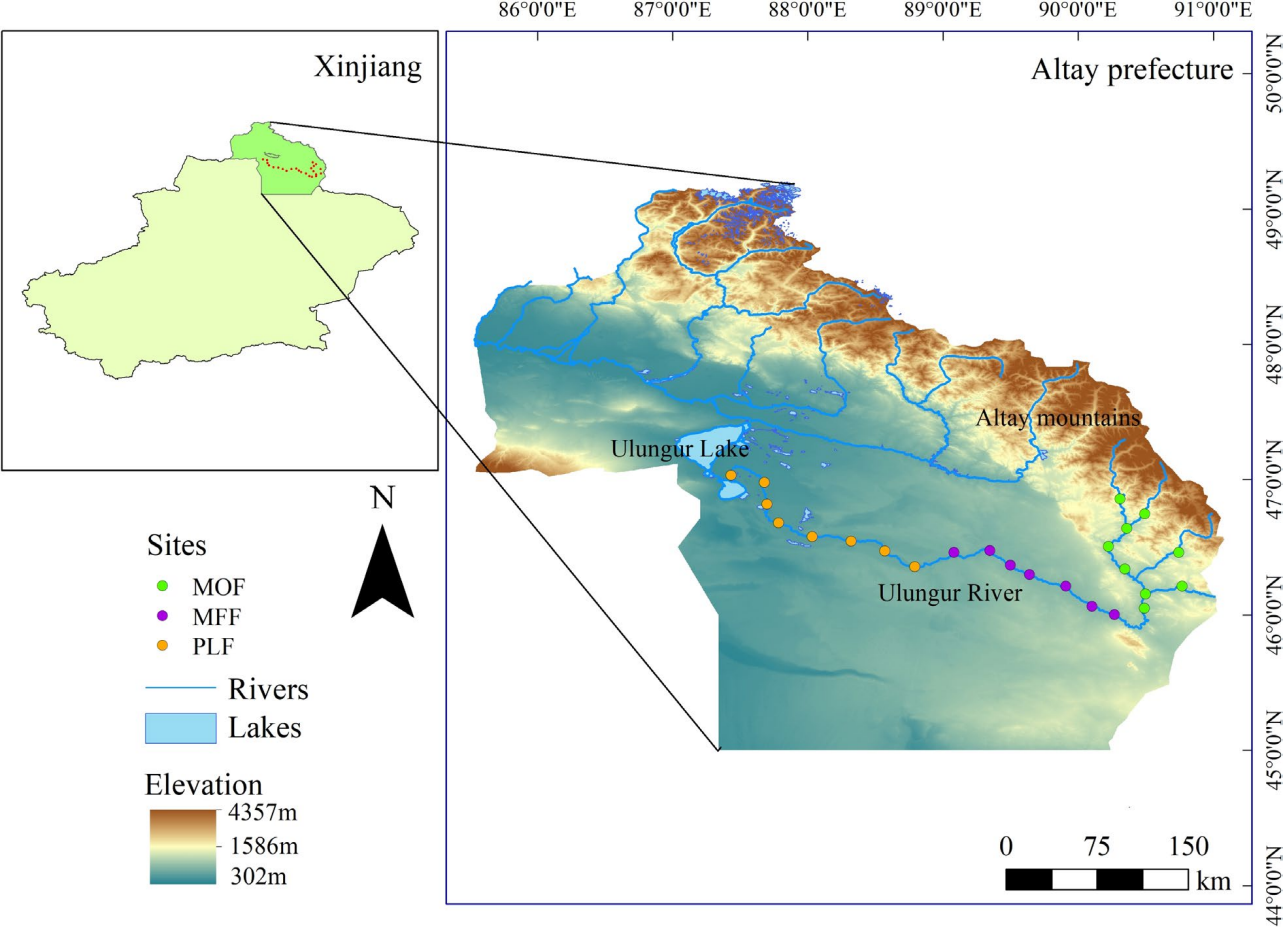
Map showing the location of study sites along the Ulungur River basin. Green dots indicate Mountain riparian forests (MOF), purple dots indicate Mountain front riparian forests (MFF), and orange dots indicate Plain riparian forests (PLF).

The study area is located in the southern Altay Mountains in northern Xinjiang, China (46°10′ ~ 47°28′ N, 87°05′ ~ 90°45′ E), within an arid region encompassing the Great Qinggil River, Small Qinggil River, Chaganguole River, Burgen River, and the Ulungur River (Figure 1). These rivers originate from the eastern Altay Mountains and together form the Ulungur River Basin, which covers approximately 37,882 km^2^ with a total river length of 811 km. The hydrological regime is primarily sustained by mountain precipitation and seasonal snowmelt. The river runoff exhibits clear seasonal characteristics, with the total runoff from May to July accounting for 65% of the annual total and substantial inter‐annual and intra‐annual variation (Bai et al. 2025).

Agricultural and pastoral irrigation, dominated by flood irrigation, represents the major water use in the basin and has contributed to soil salinization–alkalization and nutrient loss in downstream areas (Gao et al. 2020). The region has a mean annual temperature of 2.3°C, mean annual precipitation of 129.8 mm, and mean annual evaporation of 867.2 mm. The frost‐free period lasts approximately 110 ~ 130 days, and mean annual sunshine duration exceeds 3100 h. Under these climatic conditions, riparian zones support a relatively diverse herbaceous flora despite pronounced environmental constraints, with herbaceous plants being absolutely dominant (190 herbaceous species, 89.20%) (Zhang et al. 2025).

The basin exhibits a strong longitudinal gradient that provides contrasting environmental contexts along the river continuum. High‐elevation tributary reaches (> 1000 m a.s.l.) are characterized by mountain ecosystems with relatively complex vegetation structure and carbon‐rich soils. Upper mainstem reaches (725 ~ 1000 m a.s.l.), located in the piedmont transition zone, are dominated by mixed deciduous broadleaf forests with simplified structure. Lower mainstem reaches (< 725 m a.s.l.) occur in plain areas where intensive land use especially agricultural practices has resulted in pronounced soil salinization–alkalization (Liu et al. 2024). This longitudinal transition from relatively resource‐rich upstream environments to strongly stressed downstream environments provides a natural setting for examining reorganization of community assembly mechanisms along arid riparian systems.

### 2.2 Field Sampling and Species Identification

Field surveys were conducted during July and August 2024, corresponding to the peak growing season for herbaceous vegetation. Although the survey was conducted in summer and may have missed some early spring ephemeral plants, this period represents the peak growth phase for most herbaceous plants, and rare species contribute little to overall diversity patterns. Sampling sites were established along both sides of river valleys in tributaries as well as in the upper and lower reaches of the mainstem, based on vegetation representativeness and ecological relevance. To reduce spatial autocorrelation, sites were spaced approximately 20 km apart along the river network, consistent with longitudinal river classification concepts (Vannote et al. 1980).

A total of 24 sampling sites were established, including nine sites in tributaries, seven in the upper mainstem, and eight in the lower mainstem (Figure 1). Based on stream position, elevation, and associated eco‐geographical characteristics, these sites were classified into three riparian forest types following established regional classifications (B. Wang et al. 2022; Qu et al. 2025): Mountain Riparian Forest (MOF; > 1000 m a.s.l.), Mountain Front Riparian Forest (MFF; 725 ~ 1000 m a.s.l.), and Plain Riparian Forest (PLF; < 725 m a.s.l.).

At each site, four 30 m × 30 m plots were established, yielding a total of 96 plots. All plots were located close to the riverbank, within 500 m of the river. Each plot was subdivided into nine 10 m × 10 m subplots, and five 1 m × 1 m herbaceous quadrats were positioned within each plot (one central and four at the corners), resulting in 480 quadrats in total. For each quadrat, species identity, individual abundance, plant height, and percent cover were recorded, along with basic environmental information such as elevation and forest type. Plant height was measured with a ruler, abundance was recorded as the number of above‐ground stems, and percent cover was visually estimated as a continuous percentage (0% ~ 100%). All surveys were conducted by the same person to minimize subjective bias.

All plant species were initially identified in the field and subsequently standardized to contemporary taxonomic systems prior to analysis: APG IV for angiosperms, PPG I for ferns, and the Christenhusz system for gymnosperms (Christenhusz et al. 2011; APG IV 2016; PPG I 2016). To identify halophytic species, all herbaceous taxa were cross‐referenced with the Germplasm Resources of Halophytes in China database (GRHC; www.grhc.sdnu.edu.cn). Woody species recorded in the plots are listed in Table S3, but they were excluded from the main analyses due to their low abundance, scarce seedlings, and because soil sampling was specifically designed for herbaceous plants.

### 2.3 Treatment of Soil Factors

Soil samples were collected from each plot using a five‐point sampling design. At each point, surface soil (0 ~ 12 cm) was sampled to represent the primary rooting zone of herbaceous plants. Subsamples from each plot were homogenized, air‐dried, and passed through a 2 mm sieve prior to laboratory analysis. The following soil variables were measured to capture key dimensions of environmental filtering: soil organic carbon (SOC), total nitrogen (TN), and total phosphorus (TP) represent major soil nutrient pools that influence herbaceous plant growth; cation exchange capacity (CEC) and pH are key indicators of soil salinization–alkalization.

The analysis of all soil factors followed the corresponding standard procedures. Specifically, soil organic carbon (SOC) was measured using the potassium dichromate‐sulfuric acid oxidation method (NY/T 1121.6‐2006). Total nitrogen (TN) was determined by the boric acid‐sulfuric acid digestion method, and total phosphorus (TP) by acid digestion followed by molybdenum‐antimony colorimetry (LY/T 1228‐2015; LY/T 1232‐2015). Cation exchange capacity (CEC) was determined using cobalt hexamine trichloride exchange and UV spectrophotometry (HJ 889‐2017). Soil pH was determined potentiometrically in the same soil‐water suspension (HJ 613‐2011; NY/T 1377‐2007). The carbon‐to‐nitrogen ratio for each sample was simultaneously calculated using the following method:

(1) $$C/N=\frac{\mathrm{SOC}}{\mathrm{TN}}$$

### 2.4 Statistical Analyses

Based on the revised species checklist and the initial survey data, two alpha diversity indices were used to characterize herb diversity: species richness (HSR) and Shannon–Wiener diversity index (HSN) (Cousins 1991; Veech et al. 2002; Spellerberg and Fedor 2003):

(2) $$\begin{array}{c} \mathrm{HSR}=S\; \end{array}$$

(3) $$\begin{array}{c} \mathrm{HSN}=-\sum_{i=1}^{s}P_{i}\;\ln P_{i} \end{array}$$

where *S* = number of species, *P*_*i*_ = relative cover of species *i*.

For subsequent statistical analysis, the species richness of halophyte plants (HaloSR) was also statistically analyzed, with the calculation method as follows:

(4) $$\begin{array}{c} \text{HaloSR}=H \end{array}$$

where *H* = number of halophytes. Halophyte species richness (HaloSR) was calculated as the count of halophyte species per plot.

The data types used for each analysis are summarized in Table S2. Species richness, the Shannon–Wiener diversity index, and halophyte richness were calculated for each plot using R based on the initial survey data. All statistical analyses were conducted with the aim of ensuring robustness and reproducibility of the results. The abbreviations for key variables and some analytical methods used in this study, along with their data sources, are presented in Table 1. Prior to data analysis, all key variables were subjected to Shapiro–Wilk normality tests as a preprocessing step. The results of these normality tests are shown in Table 2.

**TABLE 1:** Basic characteristics of all forest types in the study area.

Abbreviation	Full name	Dimension	Supplementary comments	Data sources
MOF	Mountain riparian forest	None	Upper reaches	River section group
MFF	Mountain front riparian forest	None	Middle reaches	River section group
PLF	Plain riparian forest	None	Lower reaches	River section group
HSR	Total herbaceous species richness	1	Based on 30 × 30 m plot	Number of species count
HSN	Herbaceous Shannon–Wiener index	1	Based on 30 × 30 m plot	Percentage coverage of species
HaloSR (Halo)	Halophytic species richness	1	Based on 30 × 30 m plot	Number of halophyte count
ELE	Elevation	m	Based on site	Topographic information
SOC	Soil organic carbon	g/kg	Based on 30 × 30 m plot	Soil test
TN	Total nitrogen	g/kg	Based on 30 × 30 m plot	Soil test
CN (C/N)	Carbon‐to‐nitrogen ratio	1	Based on 30 × 30 m plot	Soil test
TP	Total phosphorus	g/kg	Based on 30 × 30 m plot	Soil test
CEC	Cation exchange capacity	cmol^+^/kg	Based on 30 × 30 m plot	Soil test
pH	Potential of hydrogen	1	Based on 30 × 30 m plot	Soil test
NMDS	Non‐metric multidimensional scaling	—	Based on river section	Percentage coverage of species

**TABLE 2:** Normality test results for soil properties, environmental variables, and plant diversity indices.

Variable	Shapiro–Wilk *W*	Shapiro–Wilk *p*	Skewness	Kurtosis
pH	0.832	< 0.001***	−1.669	5.847
TP	0.887	< 0.001***	1.378	5.050
SOC	0.852	< 0.001***	1.846	8.155
TN	0.872	< 0.001***	1.751	7.959
C/N	0.990	0.673	0.108	2.591
CEC	0.978	0.108	0.542	3.596
ELE	0.950	< 0.01**	0.232	1.923
HSR	0.984	0.289	0.039	2.456
HSN	0.987	0.474	−0.355	3.472
HaloSR	0.937	< 0.001***	0.732	2.932

* *p* < 0.05.

** *p* < 0.01.

*** *p* < 0.001.

Based on the normality test results, for variables conforming to a normal distribution, one‐way ANOVA followed by Tukey's HSD test was used; for variables not conforming to a normal distribution, the non‐parametric Kruskal–Wallis test followed by Dunn's post hoc comparisons was applied. Using herbaceous species cover data, non‐metric multidimensional scaling (NMDS) based on a Bray–Curtis dissimilarity matrix was performed with the vegan package in R to visualize differences in community composition among river segments. Permutational multivariate analysis of variance (PERMANOVA) was used to test the statistical significance of differences between groups, and the betadisper function was used to test the homogeneity of multivariate dispersions among groups.

To explore the effects of environmental factors on plant diversity, Spearman's rank correlation analysis was first used to evaluate monotonic relationships between each environmental factor and the diversity metrics. Based on this, a minimum common set of variables that were significantly correlated (*p* < 0.05) with at least one response variable was selected for subsequent simple linear regression fitting. Using this variable set, scatter plots were generated in Origin 2024 with herbaceous species richness (HSR), Shannon–Wiener index (HSN), and halophyte species richness (HaloSR) as response variables and the corresponding environmental variables as predictors. General linear regression fitting was performed using Simple Fit 4.30 software to visually illustrate the relationships between key environmental factors and plant diversity metrics.

On this basis, to further quantify the independent contributions of environmental factors to plant diversity while controlling for interactions among variables, a generalized linear model (GLM) was used. Before performing GLM analysis, variance inflation factor (VIF) analysis was conducted on all independent variables to ensure model feasibility. All soil variables were first analyzed, and then, based on preliminary results, elevation and the selected soil variables were included together in the analysis. The results are shown in Table 3.

**TABLE 3:** Variance inflation factor diagnostics for candidate.

Variable	VIF	Supplementary comments
pH	1.31	Only soil factors before generalized linear model
TP	1.11	Only soil factors before generalized linear model
SOC	**97.78**	Only soil factors before generalized linear model
TN	**82.13**	Only soil factors before generalized linear model
CN	4.18	Only soil factors before generalized linear model
CEC	1.59	Only soil factors before generalized linear model
ELE	1.69	Factors participating in generalized linear model
pH	1.79	Factors participating in generalized linear model
TP	1.16	Factors participating in generalized linear model
SOC	2.61	Factors participating in generalized linear model
CN	2.86	Factors participating in generalized linear model
CEC	1.58	Factors participating in generalized linear model

*Note:* Bold values indicate variance inflation factor (VIF) greater than 5.

Based on the variable selection results, total nitrogen (TN) was excluded from subsequent multivariate analyses. Herbaceous species richness (HSR), Shannon–Wiener index (HSN), and halophyte species richness (HaloSR) were used as response variables, and the predictor variables included elevation (ELE), pH, total phosphorus (TP), soil organic carbon (SOC), carbon‐to‐nitrogen ratio (CN), and cation exchange capacity (CEC). All variables were *Z*‐score standardized (mean = 0, standard deviation = 1) prior to analysis to allow direct comparison of regression coefficients. Because the standardized response variables were continuous, all models were fitted using a Gaussian family. Model parameters were estimated using maximum likelihood estimation. The significance of each predictor was assessed using Type III likelihood ratio tests, implemented via the Anova function in the R package car, to evaluate the independent contribution of each variable while controlling for all other variables. Model diagnostics were performed using the R package performance, including tests for residual normality (Shapiro–Wilk test), variance inflation factor (VIF), and Cook's distance, to ensure that model assumptions were met. To maintain model parsimony, non‐linear trends were examined only through scatter plots and quadratic regression, and only linear terms were retained in the final GLM.

To complement the assessment of the relative importance of each predictor for plant diversity and to test the robustness of the GLM results, random forest regression was performed. HSR, HSN, and HaloSR were used as response variables, with ELE, pH, TP, SOC, CN, and CEC as predictors. Using the original data, random forest models were constructed with the randomForest package in R, setting the number of trees to 500 (ntree = 500) and using importance = TRUE to calculate variable importance. Variable importance was measured as the percentage increase in mean squared error (%IncMSE): the increase in prediction mean squared error (MSE) when the observed values of a given variable are randomly permuted. A larger increase indicates a higher contribution of that variable to model prediction accuracy, that is, greater importance. The random forest analysis served as a complement to the GLM results, helping to identify key predictors for each response variable and to compare variable importance between the two approaches.

Basic data processing and analysis were performed in IBM SPSS Statistics 27 (SPSS Inc., Chicago, IL, USA), including data standardization, normality testing, and analysis of variance (ANOVA). Correlation analysis and visualization were carried out in Origin 2024 using the Correlation Plot 1.31 tool. Higher‐order multivariate analyses, including non‐metric multidimensional scaling (NMDS), generalized linear models (GLM), and random forest regression, were conducted in the R 4.4.3 environment. All result figures in this study were generated using Origin 2024 to ensure consistency between analytical workflows and visualization.

## 3 Results

### 3.1 Environmental Soil Factors

**FIGURE 2:**
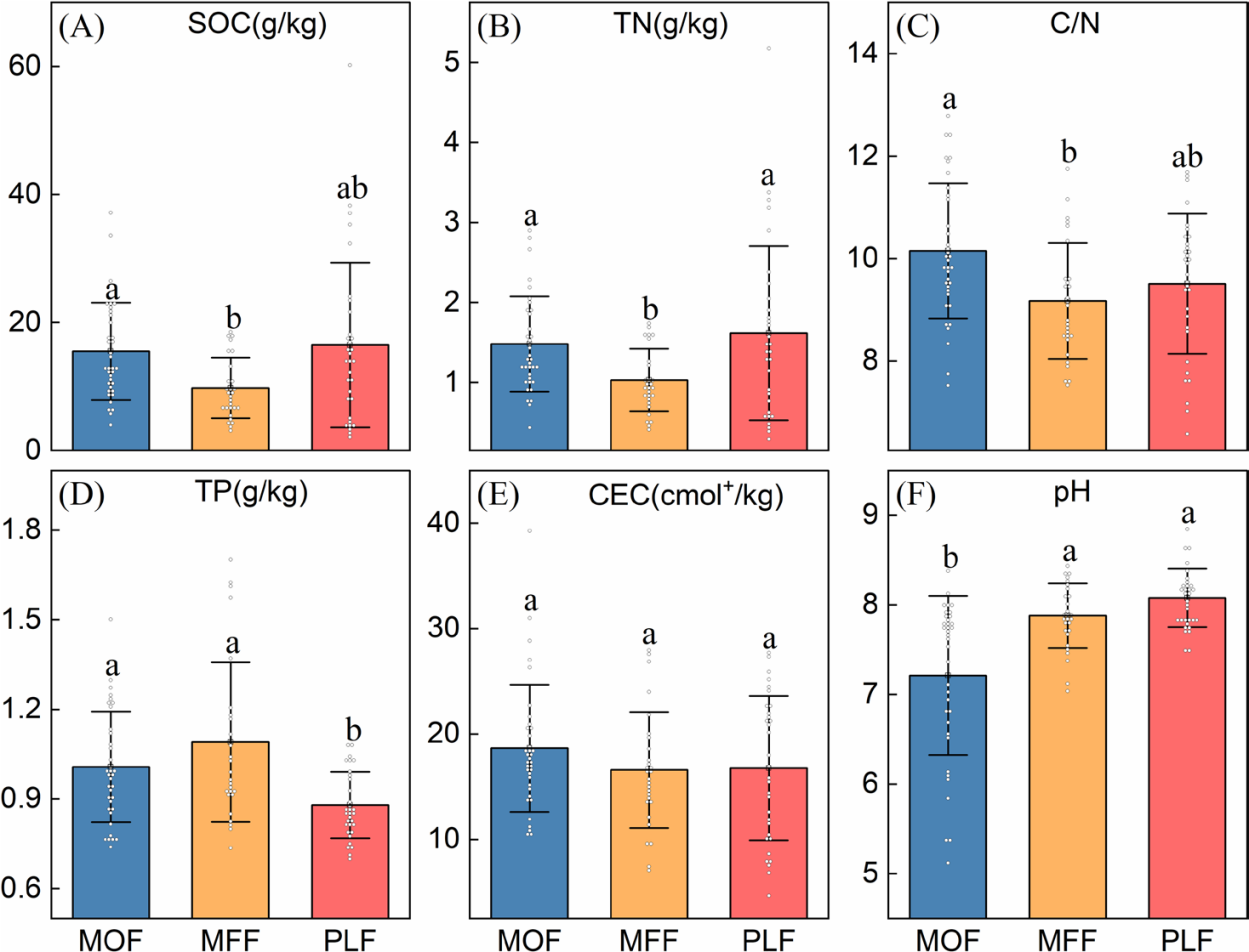
Box plots showing the distribution of soil properties across three river segments: MOF, MFF, and PLF. Panels represent (A) soil organic carbon (SOC), (B) total nitrogen (TN), (C) carbon‐to‐nitrogen ratio (C/N), (D) total phosphorus (TP), (E) cation exchange capacity (CEC), and (F) pH. The boxes indicate the interquartile range (IQR), the horizontal line inside each box represents the median, and the whiskers extend to 1.5× IQR beyond the quartiles. Points outside the whiskers are outliers. Different letters above the boxes indicate significant differences between groups at *p* < 0.05 based on Kruskal–Wallis tests followed by Dunn's post hoc comparisons with Bonferroni correction. The sample sizes are *n* = 36 for MOF, *n* = 28 for MFF, and *n* = 32 for PLF.

Soil properties exhibited pronounced spatial variation along the river continuum (Figure 2). Soil nutrient contents exhibited a pattern that was not synchronized with the salinization–alkalization stress gradient. SOC content was highest in the downstream reaches (PLF), but did not differ significantly between downstream and upstream (MOF) or midstream (MFF). Only upstream (MOF) showed significantly higher SOC than midstream (MFF). The distribution pattern of total nitrogen (TN) was highly similar to that of SOC, except that both upstream and downstream had significantly higher TN than midstream. The pattern of significant differences in the carbon‐to‐nitrogen ratio (CN) was identical to that of SOC, except that upstream values were slightly higher than downstream. Meanwhile, soil pH in the downstream reaches peaked and was significantly higher than that in the upstream reaches. Whereas total phosphorus (TP) content decreased significantly to its lowest level in the downstream, forming a multidimensional stress environment of soil salinization–alkalization and phosphorus deficiency. Cation exchange capacity (CEC) showed no significant differences among river segments. The specific ranges of soil factors for each river segment are presented in Table 4, and the classification of soil stress intensity based on TP, TN, and pH is provided in Table S4.

**TABLE 4:** Range of soil variables by river segment.

Range	SOC (g/kg)	TN (g/kg)	CN	TP (g/kg)	CEC (cmol^+^/kg)	pH
MOF	3.97 ~ 37.38 (33.41)	0.43 ~ 2.92 (2.49)	7.52 ~ 12.79 (5.28)	0.74 ~ 1.51 (0.77)	10.46 ~ 39.39 (28.93)	5.12 ~ 8.42 (3.3)
MFF	3.09 ~ 19.05 (15.96)	0.41 ~ 1.78 (1.37)	7.53 ~ 11.76 (4.24)	0.74 ~ 1.71 (0.98)	7.07 ~ 27.95 (20.88)	7.03 ~ 8.47 (1.44)
PLF	2.12 ~ 60.28 (58.15)	0.29 ~ 5.22 (4.92)	6.57 ~ 11.7 (5.13)	0.7 ~ 1.09 (0.39)	4.66 ~ 27.79 (23.13)	7.49 ~ 8.88 (1.39)

*Note:* Values are presented as minimum ~ maximum, with the range (max − min) in parentheses. For detailed distributions (median, quartiles, outliers), see Figure 2.

### 3.2 General Herbaceous Species Diversity and Halophytic Composition

**FIGURE 3:**
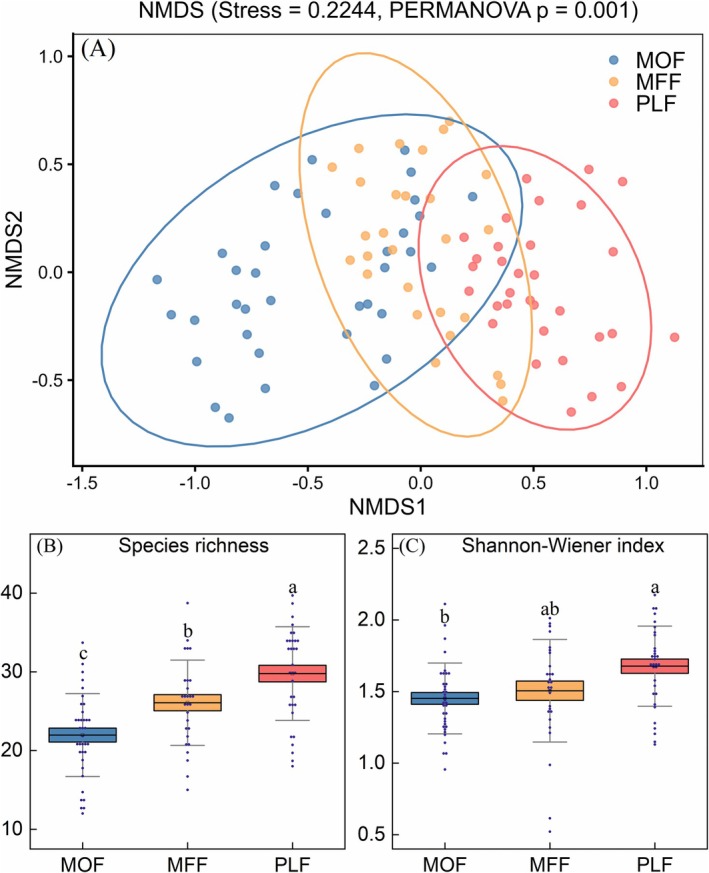
(A) Non‐metric multidimensional scaling (NMDS) ordination of herbaceous species composition. Blue dots: MOF, *n* = 36; orange dots: MFF, *n* = 28; red dots: PLF, *n* = 32. The stress value of the NMDS ordination is 0.224. Mean Bray–Curtis dissimilarity: MOF = 0.569, MFF = 0.536, PLF = 0.530. (B) Herbaceous species richness (HSR) across the three river segments. (C) Herbaceous Shannon–Wiener index (HSN) across the three river segments. In (B) and (C), different lowercase letters indicate significant differences among segments at *p* < 0.05 based on one‐way ANOVA followed by Tukey's HSD post hoc test.

In total, 214 herbaceous plant taxa (212 species, one subspecies, and one variety) were recorded across the study area (Table S1). Non‐metric multidimensional scaling (NMDS) ordination combined with PERMANOVA revealed significant differences in community composition among upstream, midstream, and downstream river segments (*p* = 0.001) (Figure 3A). Samples from the three segments formed clearly separated clusters in ordination space, with upstream communities showing the greatest within‐segment dispersion, indicating pronounced compositional heterogeneity (mean Bray–Curtis dissimilarity: MOF = 0.569, MFF = 0.536, PLF = 0.530; *F* = 7.588; *p* = 0.001).

Species richness differed significantly among all river segments (Figure 3B), with the downstream reaches (PLF) having the highest local species richness and the upstream reaches (MOF) the lowest. Similarly, the Shannon–Wiener index also peaked in the downstream reaches and was lowest in the upstream reaches (Figure 3C). However, the upstream Shannon–Wiener index was significantly lower than that of the downstream, whereas the midstream (MFF) did not differ significantly from either the upstream or the downstream. This pattern is consistent with the NMDS results, indicating that diversity patterns along the river exhibit pronounced spatial scale‐dependent differences.

**FIGURE 4:**
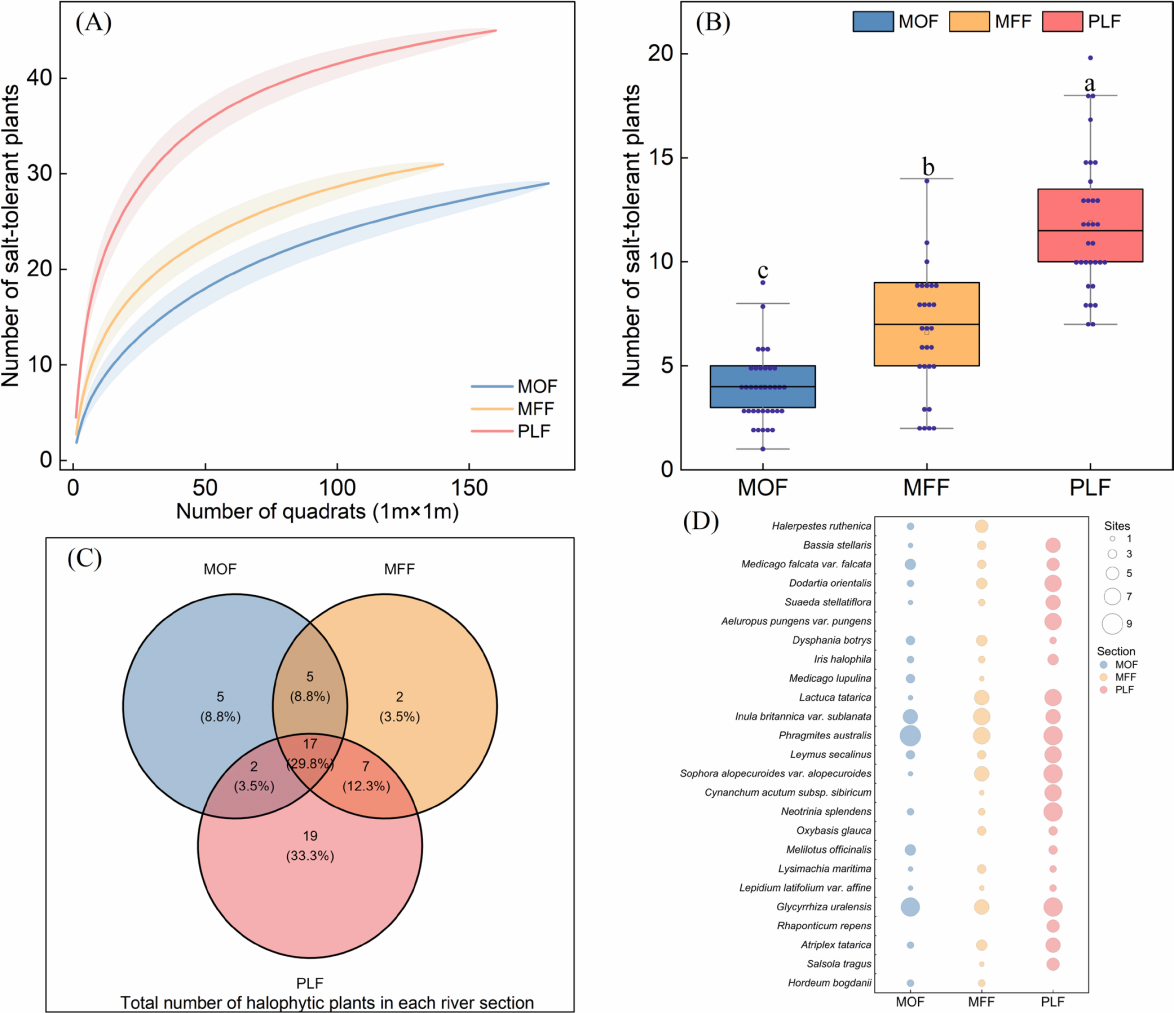
(A) Sample‐based rarefaction curves of halophyte species richness for the three river segments. The curves show the expected number of halophyte species as a function of the number of 1 m × 1 m quadrats sampled, based on 100 random permutations. Shaded areas represent 95% confidence intervals. (B) Halophytic species richness at the plot scale in each river segment. Different lowercase letters indicate significant differences among segments at *p* < 0.05 based on Kruskal–Wallis tests followed by Dunn's post hoc comparisons with Bonferroni correction. (C) Venn diagram showing the total number of halophytic species shared and unique to each river segment: MOF (blue), MFF (orange), and PLF (red). Overlap sizes indicate species co‐occurrence among segments. (D) Distribution of the top 25 dominant halophytic species across the three river segments. Bubble size is proportional to the occurrence frequency of each species across plots within a segment; larger bubbles indicate higher occurrence. Colors follow (C): Blue for MOF, orange for MFF, red for PLF.

Halophytic species, representing stress‐tolerant functional strategies, displayed clear spatial differentiation. Based on the rarefaction curves, the downstream segment retains the highest halophyte species richness at equivalent quadrat sampling effort (Figure 4A); it also shows the highest plot‐scale halophyte richness (Figure 4B), the greatest number of segment‐specific species (Figure 4C), and the most frequent halophyte species (Figure 4D). Together, these patterns indicate that downstream communities are locally species‐rich but compositionally distinct, with a strong representation of stress‐tolerant taxa.

### 3.3 The Relationship Between Environmental Factors and Plant Diversity

**FIGURE 5:**
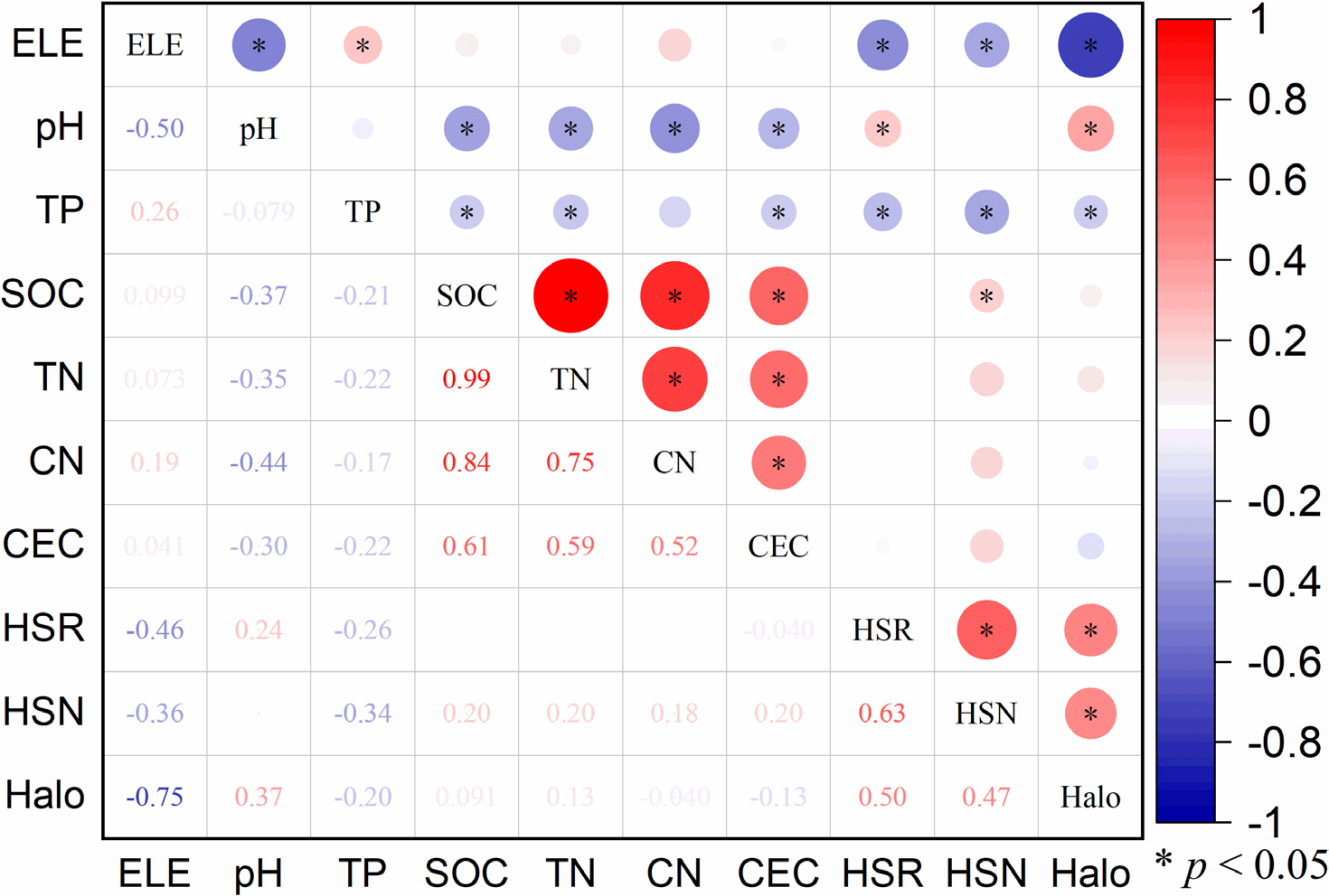
Spearman correlation matrix among environmental variables and plant diversity indices. The upper right triangle displays a heatmap of correlation significance: The size of the circle is proportional to the absolute value of the correlation coefficient, with red circles indicating positive correlations and blue circles indicating negative correlations. Asterisks (*) within circles denote statistically significant correlations (*p* < 0.05); absence of an asterisk indicates non‐significance. The lower left triangle presents the numerical values of the correlation coefficients. All variable abbreviations are defined in Table 1.

Spearman's rank correlation was first used to preliminarily assess the effects of elevation and soil variables on plant diversity. The results (Figure 5) showed that species richness was significantly negatively correlated with elevation (*r* = −0.46) and total phosphorus (TP; *r* = −0.26), and significantly positively correlated with pH (*r* = 0.24). The Shannon–Wiener index was significantly negatively correlated with elevation (*r* = −0.36) and TP (*r* = −0.34), and significantly positively correlated with soil organic carbon (SOC; *r* = 0.20). Halophyte richness was significantly negatively correlated with elevation (*r* = −0.75) and TP (*r* = −0.20), and significantly positively correlated with pH (*r* = 0.37).

**FIGURE 6:**
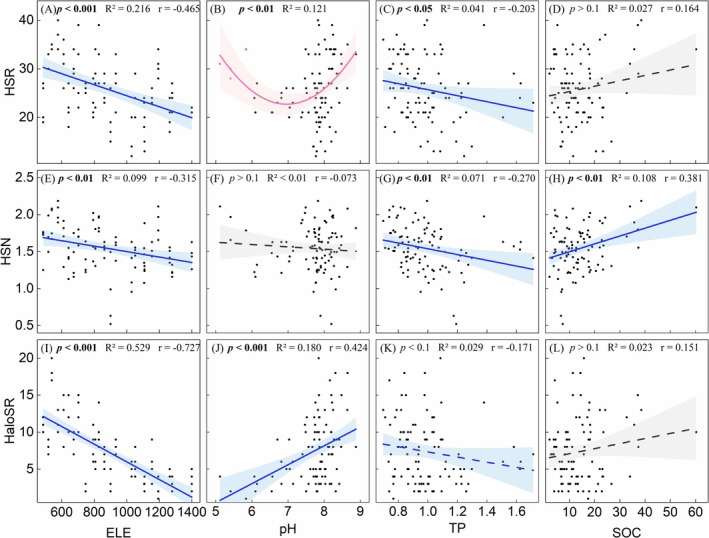
Linear regression scatterplots showing the relationships between selected environmental predictors and three plant diversity metrics. Panels are arranged in a three‐row by four‐column grid. Rows (response variables): (A–D) HSR, (E–H) HSN, and (I–L) HaloSR. Columns (predictors): (A, E, I) ELE, (B, F, J) pH, (C, G, K) TP, and (D, H, L) SOC. The predictor set represents the minimum common set of variables that showed a significant Spearman correlation with at least one of the three response variables. All variable abbreviations are defined in Table 1. In each panel, the solid line represents the ordinary least‐squares linear regression fit, with the shaded band indicating the 95% confidence interval. The corresponding *R* ^2^, *p*‐value and Pearson's *r* are shown in the upper corner of each plot. Sample size is *n* = 96 plots for all analyses.

General linear regression fitting visually illustrated the response of diversity variables to key soil factors (Figure 6). The relationship between all three diversity indices and elevation was highly consistent. With increasing elevation (400 ~ 1400 m), total species richness (HSR) (*p* < 0.001), Shannon–Wiener index (HSN) (*p* < 0.01), and halophyte richness (HaloSR) (*p* < 0.001) all significantly decreased (*r* < 0). In contrast, the relationship between the three diversity indices and pH was the most complex. HSR showed a significant non‐linear relationship with pH (*p* < 0.01). As pH increased (5.0 ~ 9.0), HSR first decreased and then increased. HSN showed no significant relationship with pH (*p* > 0.1) but was generally negatively correlated (*r* < 0). HaloSR was significantly positively correlated with pH (*p* < 0.001), increasing steadily with rising pH (5.0 ~ 9.0; *r* > 0). The response of the three diversity indices to TP was relatively consistent. With increasing TP (0.6 ~ 1.8 g/kg), HSR (*p* < 0.05) and HSN (*p* < 0.01) both significantly decreased (*r* < 0). HaloSR showed no significant correlation with TP but exhibited a decreasing trend with increasing TP (*p* < 0.1, *r* < 0). SOC had a significant effect only on HSN. With increasing SOC (0 ~ 60 g/kg), HSN increased steadily (*p* < 0.01, *r* > 0). HSR and HaloSR showed no significant relationship with SOC (*p* > 0.1) but were generally positively correlated (*r* > 0).

**FIGURE 7:**
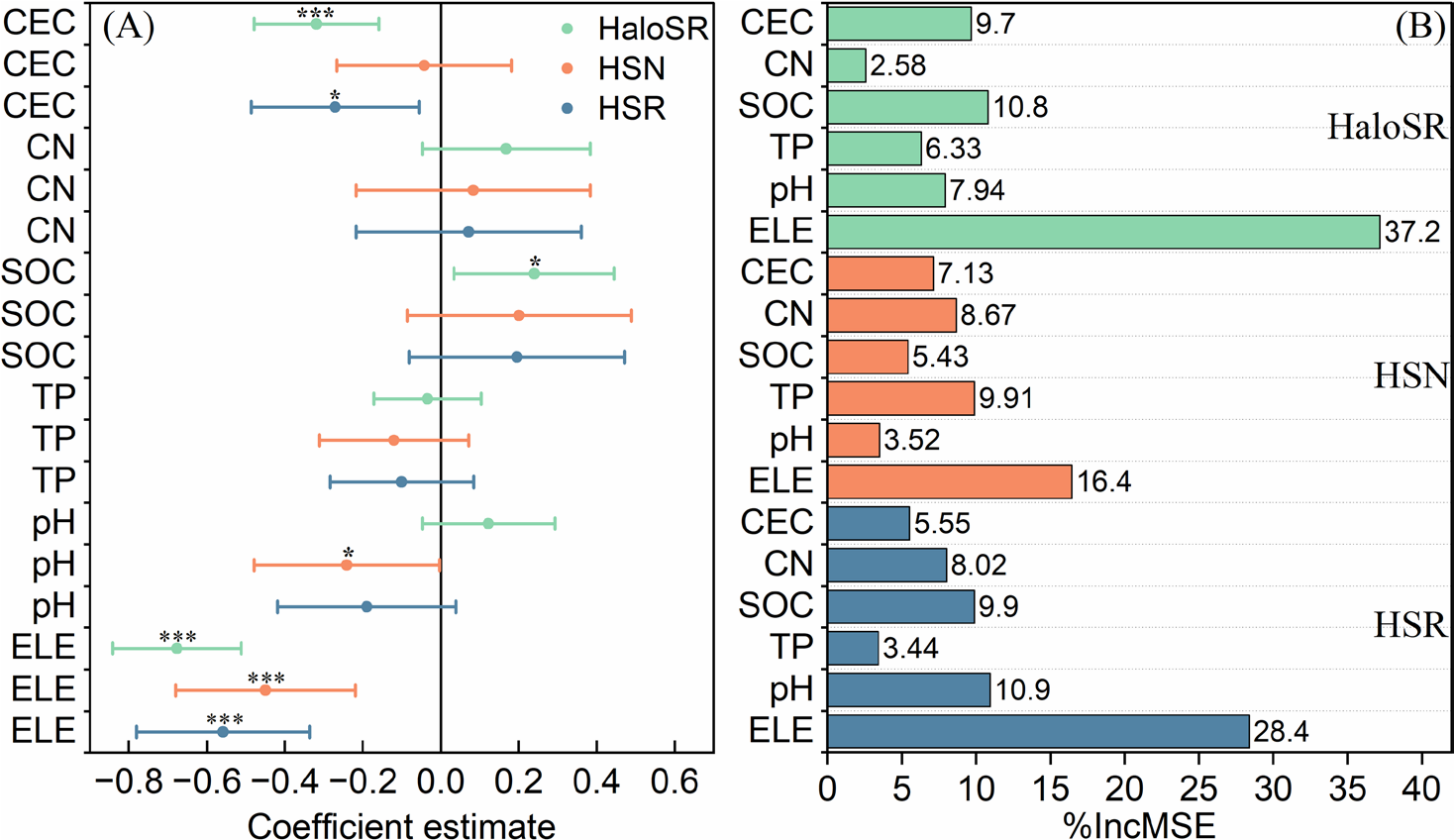
(A) Forest plots showing the standardized effect sizes (coefficients with 95% confidence intervals) from generalized linear models (GLM) examining the relationships between environmental predictors (ELE, pH, TP, SOC, CN, CEC) and three plant diversity metrics (HaloSR, HSN, HSR). All variable abbreviations are defined in Table 1. All predictors were standardized (mean = 0, SD = 1) prior to analysis to allow direct comparison of effect magnitudes. Significance levels: *p < 0.05, **p < 0.01, ***p < 0.001. Error bars represent 95% confidence intervals. (B) Variable importance plots from random forest regression models for the same three diversity metrics. Models were fitted using 500 trees, with the percentage increase in mean squared error (%IncMSE) used as the importance measure. Higher %IncMSE values indicate greater contribution of a predictor to model predictive accuracy. For each metric, all six environmental predictors were included as independent variables.

The results of generalized linear models (GLM) revealed the independent effects of environmental factors on the three plant diversity metrics (Figure 7A and Table 5). The adjusted *R* ^2^ values were 0.278 for HSR, 0.220 for HSN, and 0.600 for HaloSR, indicating that the selected predictors explained the highest proportion of variance in halophyte richness. For HSR, elevation and cation exchange capacity (CEC) showed significant independent effects. The standardized coefficient of elevation was −0.558 (*p* < 0.001), indicating that HSR significantly decreased with increasing elevation, while the coefficient of CEC was −0.271 (*p* < 0.05), also a negative effect. Soil pH, TP, SOC, and carbon‐to‐nitrogen ratio (CN) did not contribute significantly (*p* > 0.05). For HSN, elevation also had a significant negative effect (*β* = −0.450, *p* < 0.001), and pH had a coefficient of −0.241 (*p* < 0.05), indicating that HSN decreased with increasing pH. The remaining predictors (TP, SOC, CN, CEC) were not significant (*p* > 0.05). For HaloSR, elevation (*β* = −0.677, *p* < 0.001), SOC (*β* = 0.240, *p* < 0.05), and CEC (*β* = −0.319, *p* < 0.001) were all significant predictors. SOC had a positive effect, whereas elevation and CEC had negative effects. pH, TP, and CN were not significant (*p* > 0.05). Overall, elevation was the strongest common negative predictor for all diversity metrics, while the significant effects of soil properties (CEC, SOC, pH) varied among the different diversity metrics. Model diagnostics indicated that residuals of all models approximately satisfied the normality assumption (Shapiro–Wilk test *p* > 0.05), and variance inflation factor (VIF) values were all below 3, indicating no severe multicollinearity (Table 6).

**TABLE 5:** Standardized coefficients (*β*) from generalized linear models for the three plant diversity metrics.

Predictor	ELE	pH	TP	SOC	CN	CEC
HSR	−0.558***	−0.190	−0.100	0.195	0.072	−0.271*
HSN	−0.450***	−0.241*	−0.120	0.201	0.083	−0.042
HaloSR	−0.677***	0.123	−0.034	0.240*	0.168	−0.319***

* *p* < 0.05.

** *p* < 0.01.

*** *p* < 0.001.

**TABLE 6:** Generalized linear models fit and diagnostics for three diversity metrics.

Response	*R* ^2^	Adj *R* ^2^	*n*	Residual S–W *p*‐value	VIF
HSR	0.323	0.278	96	0.533	1.164 ~ 2.865
HSN	0.270	0.220	96	0.076	1.164 ~ 2.865
HaloSR	0.625	0.600	96	0.080	1.164 ~ 2.865

*Note:* All models used Gaussian distribution with standardized predictors (*n* = 96). Residual normality was assessed by Shapiro–Wilk test; variance inflation factor (VIF) ranges are shown (all VIF < 3).

Abbreviations: adj. *R* ^2^ = adjusted *R* ^2^; HaloSR = halophytic species richness; HSN = herbaceous Shannon–Wiener index; HSR = total herbaceous species richness; *R* ^2^ = coefficient of determination.

Random forest regression further assessed the relative importance of each environmental factor for plant diversity (Figure 7B). Overall, elevation showed the highest %IncMSE (percentage increase in mean squared error) for all three response variables, consistent with the GLM results, indicating that elevation was the strongest common predictor. For HSR, in addition to elevation, pH and SOC also showed high importance, although only elevation and CEC were significant in the GLM, suggesting that the importance of some variables may be expressed through non‐linear or interactive effects. For HSN, TP and CN had higher importance than other soil factors, whereas only elevation and pH were significant in the GLM. For HaloSR, the importance of elevation was most prominent, followed by SOC and CEC, consistent with the significant effects of all three in the GLM (Figure 7A). Overall, the random forest results corroborated the GLM conclusions, supporting elevation as a core driver of diversity variation while revealing differences in the importance of soil factors among diversity metrics.

## 4 Discussion

This study documents an unexpected pattern of high local plant diversity under intense environmental stress in the downstream reaches of an arid riparian system. By integrating multivariate analyses including NMDS, GLM and random forest, results show that this pattern is associated with a pronounced reorganization of environmental filtering regimes along the river continuum. Specifically, riparian position primarily structured species turnover. Elevation, as a common dominant factor, exerted significant negative effects on total herbaceous species richness (HSR), Shannon–Wiener index (HSN), and halophyte richness (HaloSR). In contrast, soil properties such as pH, SOC, and CEC showed divergent effects on different diversity metrics, with SOC and CEC having independent significant positive effects on HaloSR. Results from random forest further revealed that pH and total phosphorus (TP) may influence diversity through non‐linear or interactive effects, suggesting that severe and spatially heterogeneous stress environments can sustain locally diverse herbaceous communities via multidimensional filtering.

Typical ecological theory generally predicts declining biodiversity with increasing abiotic stress (Le Bagousse‐Pinguet et al. 2017). Frameworks such as the Intermediate Disturbance Hypothesis and the River Continuum Concept (RCC) emphasize gradual environmental change and predict peak diversity under moderate conditions, often in mid‐reach river segments (Lite et al. 2005; Surmacz et al. 2024). In contrast, our results indicate that in arid riparian systems, the highest local diversity occurs in the downstream reaches subject to the most intense soil salinization–alkalization and phosphorus stress, where pH reached up to 8.88 and TP as low as 0.70 g/kg (Li et al. 2024). This pattern is broadly consistent with the Stress‐Gradient Hypothesis (SGH), which proposes that severe stress can reduce competitive exclusion and alter species coexistence mechanisms (Bertness and Callaway 1994; de Jonge et al. 2021). The U‐shaped (first decreasing then increasing) rather than classical hump‐shaped (unimodal) relationship between pH and HSR (Chytrý et al. 2007; Crespo‐Mendes et al. 2019) further illustrates the complexity of stress‐diversity relationships. Our findings show that the internal structure and heterogeneity of the stress landscape itself play a critical role in shaping diversity patterns.

The high diversity observed downstream is likely associated with interactions among multidimensional heterogeneous stressors. The combination of soil salinization–alkalization (pH 7.49 ~ 8.88) and phosphorus scarcity (TP 0.70 ~ 1.09 g/kg) imposes multiple constraints on plant survival and performance. Notably, the overall background stress of pH and TP was highly consistent, with the downstream reaches having the smallest ranges for both pH and TP among the three segments. In contrast, the range of SOC was largest, forming a special pattern of uniform soil salinization–alkalinity and phosphorus stress but heterogeneous local carbon pools. This implies that no single optimal trait strategy is sufficient downstream, allowing species with different tolerance trade‐offs to coexist (Walker 2024). This apparent expansion of viable niche space aligns with trait‐based coexistence theory, whereby stress‐driven niche differentiation promotes species richness under severe conditions (Pérez‐Ramos et al. 2019). Although SOC was relatively high downstream and had a significant positive effect on halophyte richness, this relationship may not directly reflect increased resource availability. In soil saline‐alkaline environments, organic carbon accumulation is more likely a result of inhibited microbial decomposition (de Nijs and Cammeraat 2020), rather than simple nutrient enrichment, distinguishing these habitats from resource‐rich systems in humid environments (Mou et al. 2021). Meanwhile, our random forest results indicated a high importance of downstream pH for HSR and TP for HSN, suggesting possible non‐linear or interactive effects, exemplified by the parabolic relationship between pH and HSR. Against a background of high salinity‐alkalinity and low phosphorus, plants may therefore rely on additional strategies to acquire and utilize specific carbon resources efficiently, further promoting functional trait divergence among species (Li et al. 2023).

Patterns of community composition further support this interpretation. NMDS ordination showed that upstream plots were most spatially dispersed, and PERMDISP tests further confirmed that multivariate dispersion among upstream quadrats was significantly higher than downstream, consistent with the highly scattered pH distribution upstream. Together with the lower local α diversity in upstream reaches (Figure 3B), this pattern suggests that high upstream heterogeneity may arise from habitat fragmentation or rapid changes in local environmental gradients (Zhang et al. 2023). In contrast, although the downstream overall habitat is more homogeneous, it compensates for lower beta diversity by having higher local α diversity (especially of halophytes) at the plot scale. Fine‐scale differences in salinity and phosphorus availability within microhabitats may provide niche differentiation space for species with different tolerance trade‐offs (Shen et al. 2024), leading to enhanced species coexistence within downstream plots.

The reorganization of dominant plant functional strategies along the river continuum, inferred from the increasing dominance of halophytes (as proxies for stress‐tolerant species) downstream and the corresponding decline of non‐halophytic species upstream, provides additional biological context for these patterns. This reorganization is qualitatively consistent with predictions of CSR strategy theory (Grime 1974), although direct trait measurements would be needed to confirm this interpretation. Dominant halophytic taxa in downstream reaches exhibit convergent adaptations to soil salinization–alkalization and phosphorus limitation, while the presence of numerous locally distributed species suggests ongoing ecological differentiation across heterogeneous microhabitats (Martínez‐Blancas and Martorell 2020; Tysklind et al. 2020). Although these patterns are consistent with patterns often attributed to long‐term evolutionary responses to stress, here they are more parsimoniously interpreted as outcomes of ecological filtering operating across spatial gradients.

It is important to note that in this study, river segment, elevation, and soil factors are nested, making it impossible to fully separate their independent effects. The conclusions should therefore be understood as correlations rather than strict causal relationships. In addition, due to logistical constraints, we were unable to measure plant functional traits, which could provide more direct evidence for stress‐filtering mechanisms. Future studies should integrate functional trait analyses to further test the mechanisms proposed here. Of particular interest is the large variability in downstream SOC, which suggests that spatial differentiation of carbon pools may be a key driver of local species coexistence; future research combining root traits and microbial community analyses could explore this in greater depth.

Despite these limitations, overall, our findings highlight the importance of considering stress heterogeneity and spatially structured reorganization of environmental filtering when examining biodiversity patterns in arid riparian ecosystems. Rather than uniformly reducing diversity, intense environmental stress that is spatially structured and multidimensional can promote coexistence through trait differentiation and spatial turnover (Jentsch and White 2019). These insights contribute to a more nuanced understanding of biodiversity maintenance under severe conditions and underscore the need to incorporate stress heterogeneity into sustainable management of arid river systems.

## 5 Conclusion

This study shows that high local plant diversity can be maintained in downstream riparian zones of arid river systems despite intense environmental stress. This pattern is associated with a reorganization in environmental filtering mechanisms along the river continuum: from upstream species turnover dominated by resource competition and habitat heterogeneity to a multidimensional heterogeneous stress landscape in downstream reaches jointly constituted by soil salinization–alkalization and phosphorus deficiency. In this context, elevation emerged as a common dominant factor exerting a significant negative effect on diversity, while soil organic carbon (SOC) and cation exchange capacity (CEC) had independent positive effects on halophyte richness; pH and total phosphorus may indirectly influence community structure through non‐linear or interactive effects. The resulting multidimensional and spatially heterogeneous stress landscape is consistent with the promotion of functional differentiation and niche complementarity among stress‐tolerant species, thereby supporting locally diverse plant assemblages.

These findings provide empirical support for the importance of stress heterogeneity and reorganization of environmental filtering in arid riparian ecosystems, and offer a more nuanced perspective on the application of the River Continuum Concept and the Stress‐Gradient Hypothesis under severe environmental conditions. By advancing our understanding of biodiversity maintenance in arid regions, this study also has important implications for the conservation and management of riparian ecosystems facing increasing environmental pressure.

## Author Contributions

**Zidong Zhang:** data curation (equal), formal analysis (equal), investigation (equal), methodology (equal), writing – original draft (lead). **Zhifang Xue:** formal analysis (equal), investigation (equal), methodology (equal). **Tiantian Qin:** formal analysis (equal), investigation (equal), software (supporting). **Shengtianzi Dong:** conceptualization (equal), data curation (equal), investigation (equal), supervision (equal), writing – review and editing (equal). **Hanyue Wang:** conceptualization (equal), data curation (equal), funding acquisition (lead), investigation (equal), supervision (equal), writing – review and editing (equal).

## Funding

This work was supported equally by Tianchi Talent Project of Xinjiang (Grant number CZ001622); High‐level Talents Scientific Startup Project of Shihezi University (Grant number RCZK202472).

## Conflicts of Interest

The authors declare no conflicts of interest.

## Supporting information


**Table S1:** Herbaceous plant species recorded across 96 plots (24 sites) along the riparian gradient of the Ulungur River basin, showing site‐level occurrence frequency, halophyte classification, and importance values.
**Table S2:** Woody plant species occurrence across river segments.
**Table S3:** Data types used for each analysis.
**Table S4:** Classification of soil pH, total phosphorus (TP), and total nitrogen (TN) levels for assessing stress intensity.

## Data Availability

The data supporting the findings of this study are provided in the Supporting Information files accompanying this manuscript. These materials include species occurrence data, diversity indices, soil physicochemical measurements, and summary datasets used for all analyses. All data are available to editors and reviewers during the peer review process. All data used to support the conclusions of this study are available at https://doi.org/10.5281/zenodo.19064584. *Code Availability*: The codes for the analyses of this study are available at https://doi.org/10.5281/zenodo.19064584.
