# Supplementary material for: Heterogeneous Stress Driven by Environmental Filtering Maintains Plant Diversity Along an Arid Riparian Gradient
Source: Ecol Evol. 2026 Jun 10;16(6):e73745. doi: 10.1002/ece3.73745 (PMC13250630; doi:10.1002/ece3.73745)
Supplement: Supplementary file 1 — Table S1: Herbaceous plant species recorded across 96 plots (24 sites) along the riparian gradient of the Ulungur River basin, showing site‐level occurrence frequency, halophyte classification, and importance values. Table S2: Woody plant species occurrence across river segments. Table S3: Data types used for each analysis. Table S4: Classification of soil pH, total phosphorus (TP), and total nitrogen (TN) levels for assessing stress intensity. [file ECE3-16-e73745-s001.docx]

**Supplementary Table S1** Herbaceous plant species recorded across 96 plots (24 sites) along the riparian gradient of the Ulungur River basin, showing site-level occurrence frequency, halophyte classification, and importance values.

| Species (Based on APG IV) | No. of sites in mountain riparian forest (MOF) | No. of sites in mountain-front riparian forest (MFF) | No. of sites in plain riparian forest (PLF) | Halophyte status (Yes/No; based on GRHC) |
| --- | --- | --- | --- | --- |
| *Equisetum ramosissimum* subsp. *ramosissimum* | 0 | 1 | 5 | No |
| *Equisetum arvense* | 8 | 3 | 0 | No |
| *Alisma orientale* | 0 | 1 | 0 | No |
| *Butomus umbellatus* | 0 | 1 | 0 | No |
| *Triglochin palustris* | 1 | 1 | 0 | Yes |
| *Triglochin maritima* | 0 | 1 | 0 | Yes |
| *Iris halophila* | 2 | 2 | 4 | Yes |
| *Allium ramosum* | 1 | 0 | 0 | No |
| *Allium senescens* | 1 | 3 | 2 | No |
| *Asparagus neglectu* | 3 | 4 | 7 | No |
| *Juncus gracillimus* | 4 | 5 | 4 | No |
| *Blysmus sinocompressus* var. *sinocompressus* | 1 | 0 | 0 | No |
| *Carex stenocarpa* | 3 | 3 | 3 | No |
| *Carex vulpina* | 1 | 0 | 0 | No |
| *Carex cespitosa* | 1 | 0 | 0 | No |
| *Carex rugulosa* | 1 | 0 | 0 | No |
| *Carex leiorhyncha* | 1 | 0 | 0 | No |
| *Eleocharis palustris* | 3 | 2 | 1 | No |
| *Bolboschoenus maritimus* | 0 | 1 | 0 | No |
| *Bolboschoenus planiculmis* | 4 | 6 | 3 | No |
| *Schoenoplectus tabernaemontani* | 0 | 2 | 0 | No |
| *Cyperus michelianus* | 0 | 0 | 1 | No |
| *Cyperus fuscus* form. fuscus | 0 | 0 | 1 | No |
| *Pycreus sanguinolentus* form. *sanguinolentus* | 0 | 1 | 1 | No |
| *Neotrinia splendens* | 2 | 2 | 8 | Yes |
| *Bromus squarrosus* | 3 | 0 | 0 | No |
| *Bromus inermis* | 4 | 6 | 5 | No |
| *Leymus secalinus* | 3 | 3 | 7 | Yes |
| *Hordeum roshevitzii* | 5 | 4 | 5 | No |
| *Hordeum bogdanii* | 2 | 2 | 0 | Yes |
| *Triticum aestivum* | 0 | 1 | 0 | No |
| *Agrostis gigantea* | 8 | 7 | 3 | No |
| *Calamagrostis pseudophragmites* | 2 | 6 | 5 | No |
| *Calamagrostis epigeios* var. *epigeios* | 0 | 1 | 2 | Yes |
| *Lolium perenne* | 8 | 7 | 8 | No |
| *Dactylis glomerata* subsp. *glomerata* | 2 | 0 | 0 | No |
| *Poa nemoralis* subsp. *nemoralis* | 3 | 2 | 1 | No |
| *Poa pratensis* var. *pratensis* | 4 | 2 | 0 | No |
| *Poa pratensis* subsp. *angustifolia* | 0 | 1 | 0 | No |
| *Beckmannia syzigachne* var. *syzigachne* | 0 | 0 | 1 | No |
| *Alopecurus aequalis* | 6 | 4 | 0 | No |
| *Phragmites australis* | 9 | 7 | 8 | Yes |
| *Eragrostis pilosa* var. *pilosa* | 2 | 4 | 4 | No |
| *Chloris virgata* | 0 | 0 | 3 | No |
| *Aeluropus pungens* var. *pungens* | 0 | 0 | 7 | Yes |
| *Echinochloa crus-galli* var. *crus-galli* | 3 | 5 | 4 | No |
| *Setaria viridis* subsp. *viridis* | 3 | 4 | 6 | No |
| *Zea mays* | 0 | 0 | 1 | No |
| *Thalictrum simplex* var. simplex | 7 | 6 | 4 | No |
| *Clematis glauca* | 2 | 2 | 5 | No |
| *Halerpestes ruthenica* | 2 | 5 | 0 | Yes |
| *Ranunculus meyerianus* | 2 | 0 | 0 | No |
| *Tribulus terrestris* | 0 | 0 | 1 | No |
| *Zygophyllum gobicum* | 0 | 0 | 1 | No |
| *Sophora alopecuroides* var. *alopecuroides* | 1 | 6 | 8 | Yes |
| *Lotus corniculatus* var. *corniculatus* | 1 | 2 | 0 | Yes |
| *Lotus frondosus* | 2 | 0 | 0 | Yes |
| *Glycyrrhiza uralensis* | 8 | 6 | 8 | Yes |
| *Oxytropis glabra* | 0 | 1 | 1 | Yes |
| *Astragalus contortuplicatus* | 0 | 0 | 2 | Yes |
| *Astragalus tibetanus* | 1 | 0 | 0 | No |
| *Sphaerophysa salsula* | 0 | 0 | 3 | Yes |
| *Cicer microphyllum* | 1 | 0 | 0 | No |
| *Medicago falcata* var. *falcata* | 4 | 3 | 5 | Yes |
| *Medicago lupulina* | 3 | 1 | 0 | Yes |
| *Medicago sativa* | 3 | 2 | 3 | No |
| *Medicago varia* | 0 | 0 | 2 | Yes |
| *Melilotus albus* | 0 | 0 | 1 | Yes |
| *Melilotus officinalis* | 4 | 0 | 3 | Yes |
| *Trifolium fragiferum* | 2 | 0 | 0 | Yes |
| *Trifolium lupinaster* var. *lupinaster* | 3 | 0 | 0 | No |
| *Trifolium pratense* | 2 | 0 | 0 | No |
| *Trifolium repens* | 6 | 2 | 1 | No |
| *Vicia cracca* var. *cracca* | 7 | 1 | 1 | No |
| *Vicia lilacina* | 1 | 0 | 0 | No |
| *Vicia sativa* subsp. *nigra* | 7 | 3 | 1 | No |
| *Vicia tetrasperma* | 5 | 0 | 0 | No |
| *Lathyrus palustris* var. *exalatus* | 1 | 2 | 0 | No |
| *Lathyrus pratensis* | 4 | 0 | 0 | No |
| *Geum aleppicum* | 1 | 0 | 0 | No |
| *Agrimonia pilosa* var. *pilosa* | 1 | 0 | 0 | No |
| *Potentilla argentea* | 8 | 4 | 5 | No |
| *Potentilla supina* var. *supina* | 1 | 2 | 2 | No |
| *Argentina anserina* | 6 | 5 | 1 | No |
| *Sibbaldianthe bifurca* | 5 | 2 | 3 | No |
| *Alchemilla japonica* | 1 | 0 | 0 | No |
| *Cannabis sativa* | 0 | 0 | 1 | No |
| *Urtica cannabina* | 1 | 0 | 0 | No |
| *Viola altaica* | 2 | 0 | 0 | No |
| *Viola occulta* | 1 | 0 | 0 | No |
| *Viola yunnanfuensis* | 1 | 0 | 0 | No |
| *Euphorbia soongarica* | 0 | 0 | 3 | Yes |
| *Geranium divaricatum* | 2 | 0 | 1 | No |
| *Geranium albiflorum* | 2 | 0 | 0 | No |
| *Geranium pratense* | 6 | 3 | 0 | No |
| *Lythrum salicaria* | 1 | 5 | 2 | No |
| *Hibiscus trionum* | 0 | 0 | 1 | No |
| *Althaea officinalis* | 0 | 1 | 1 | No |
| *Alyssum desertorum* | 1 | 0 | 1 | No |
| *Rorippa palustris* | 0 | 2 | 0 | No |
| *Rorippa sylvestris* | 1 | 0 | 0 | No |
| *Lepidium chalepense* | 1 | 1 | 2 | No |
| *Lepidium latifolium* var. *affine* | 1 | 1 | 2 | Yes |
| *Thlaspi arvense* | 1 | 0 | 0 | No |
| *Sisymbrium loeselii* | 1 | 0 | 0 | No |
| *Limonium gmelinii* | 1 | 0 | 1 | Yes |
| *Persicaria amphibia* | 0 | 1 | 0 | No |
| *Persicaria hydropiper* | 3 | 0 | 0 | No |
| *Persicaria lapathifolia* | 0 | 0 | 1 | No |
| *Persicaria maculosa* | 2 | 5 | 2 | No |
| *Rumex acetosella* | 2 | 6 | 4 | No |
| *Rumex crispus* | 5 | 3 | 4 | No |
| *Rumex longifolius* | 2 | 0 | 0 | No |
| *Polygonum argyrocoleon* | 2 | 0 | 0 | No |
| *Polygonum aviculare* var. aviculare | 3 | 5 | 5 | No |
| *Polygonum schischkinii* | 0 | 0 | 3 | No |
| *Sagina maxima* | 1 | 3 | 0 | No |
| *Cerastium arvense* subsp. *strictum* | 1 | 0 | 0 | No |
| *Silene repens* | 4 | 0 | 0 | No |
| *Gypsophila paniculata* | 0 | 0 | 1 | Yes |
| *Gypsophila perfoliata* | 0 | 0 | 2 | Yes |
| *Suaeda physophora* | 1 | 0 | 0 | Yes |
| *Suaeda stellatiflora* | 1 | 2 | 6 | Yes |
| *Suaeda altissima* | 0 | 0 | 1 | Yes |
| *Suaeda salsa* | 0 | 0 | 1 | Yes |
| *Suaeda glauca* | 0 | 0 | 1 | Yes |
| *Suaeda prostrata* | 0 | 0 | 2 | Yes |
| *Suaeda microphylla* | 0 | 0 | 1 | Yes |
| *Salicornia europaea* | 0 | 1 | 2 | Yes |
| *Bassia stellaris* | 1 | 3 | 6 | Yes |
| *Grubovia dasyphylla* | 0 | 0 | 1 | Yes |
| *Pyankovia brachiata* | 0 | 0 | 1 | Yes |
| *Salsola tragus* | 0 | 1 | 5 | Yes |
| *Amaranthus albus* | 0 | 1 | 3 | No |
| *Amaranthus retroflexus* var. *retroflexus* | 0 | 1 | 4 | No |
| *Corispermum lehmannianum* | 0 | 0 | 3 | No |
| *Ceratocarpus arenarius* | 0 | 0 | 1 | Yes |
| *Dysphania botrys* | 3 | 4 | 2 | Yes |
| *Oxybasis glauca* | 0 | 3 | 3 | Yes |
| *Chenopodiastrum hybridum* | 1 | 6 | 5 | No |
| *Atriplex tatarica* | 2 | 4 | 6 | Yes |
| *Chenopodium acuminatum* subsp. *acuminatum* | 0 | 0 | 2 | No |
| *Chenopodium album* | 0 | 2 | 2 | No |
| *Chenopodium ficifolium* | 4 | 7 | 8 | No |
| *Lysimachia maritima* | 1 | 3 | 2 | Yes |
| *Lysimachia vulgaris* | 0 | 3 | 2 | No |
| *Rubia dolichophylla* | 1 | 1 | 3 | No |
| *Galium aparine* var. *echinospermum* | 0 | 0 | 3 | No |
| *Galium boreale* var. *boreale* | 5 | 0 | 0 | No |
| *Galium verum* var. *verum* | 2 | 1 | 0 | No |
| *Centaurium pulchellum* var. *pulchellum* | 0 | 1 | 3 | No |
| *Centaurium pulchellum* var. *altaicum* | 0 | 1 | 0 | No |
| *Apocynum venetum* | 1 | 1 | 2 | Yes |
| *Cynanchum acutum* subsp. *sibiricum* | 0 | 1 | 7 | Yes |
| *Anchusa ovata* | 0 | 1 | 0 | No |
| *Lithospermum officinale* | 0 | 0 | 1 | No |
| *Lappula patula* | 3 | 2 | 3 | No |
| *Cynoglossum viridiflorum* | 2 | 0 | 0 | Yes |
| *Cuscuta campestris* | 0 | 0 | 4 | No |
| *Convolvulus arvensis* | 1 | 3 | 7 | No |
| *Solanum nigrum* var. *nigrum* | 0 | 0 | 1 | No |
| *Solanum dulcamara* | 1 | 1 | 0 | No |
| *Veronica anagalloides* | 0 | 2 | 0 | No |
| *Pseudolysimachion spicatum* | 1 | 0 | 0 | No |
| *Plantago major* | 8 | 7 | 6 | No |
| *Plantago minuta* | 0 | 1 | 0 | Yes |
| *Plantago salsa* | 1 | 0 | 0 | Yes |
| *Hyssopus cuspidatus* var. *cuspidatus* | 1 | 0 | 0 | No |
| *Mentha canadensis* | 4 | 4 | 0 | No |
| *Scutellaria scordiifolia* var. *scordiifolia* | 1 | 1 | 0 | No |
| *Dodartia orientalis* | 2 | 4 | 7 | Yes |
| *Rhinanthus glaber* | 4 | 0 | 0 | No |
| *Synurus deltoides* | 1 | 1 | 3 | No |
| *Saussurea robusta* | 0 | 1 | 3 | Yes |
| *Saussurea salsa* | 0 | 0 | 2 | Yes |
| *Cirsium arvense* var. *integrifolium* | 4 | 6 | 3 | No |
| *Rhaponticum repens* | 0 | 0 | 5 | Yes |
| *Hieracium robustum* | 1 | 0 | 0 | No |
| *Hieracium umbellatum* | 2 | 0 | 0 | No |
| *Hieracium virosum* | 1 | 0 | 0 | No |
| *Lactuca serriola* | 2 | 5 | 2 | No |
| *Lactuca tatarica* | 1 | 6 | 7 | Yes |
| *Sonchus transcaspicus* | 2 | 4 | 0 | No |
| *Sonchus wightianus* | 2 | 7 | 5 | No |
| *Taraxacum longipyramidatum* | 0 | 0 | 1 | No |
| *Taraxacum officinale* | 8 | 7 | 4 | No |
| *Taraxacum pseudoalpinum* | 1 | 0 | 0 | No |
| *Ligularia xinjiangensis* | 2 | 0 | 0 | No |
| *Jacobaea argunensis* | 5 | 7 | 3 | No |
| *Aster altaicus* var. *altaicus* | 0 | 2 | 6 | No |
| *Erigeron acris* subsp. *politus* | 2 | 0 | 0 | No |
| *Erigeron annuus* | 1 | 0 | 0 | No |
| *Erigeron canadensis* | 1 | 0 | 0 | No |
| *Artemisia annua* | 2 | 7 | 8 | No |
| *Artemisia pubescens* var. *pubescens* | 2 | 2 | 6 | No |
| *Artemisia sieversiana* | 4 | 3 | 6 | No |
| *Artemisia dracunculus* var. *dracunculus* | 0 | 1 | 3 | No |
| *Artemisia frigida* var. frigida | 0 | 0 | 4 | No |
| *Artemisia lavandulifolia* | 2 | 2 | 3 | No |
| *Artemisia vulgaris* var. vulgaris | 2 | 1 | 0 | No |
| *Seriphidium terrae-albae* | 2 | 0 | 5 | No |
| *Achillea millefolium* | 3 | 0 | 0 | No |
| *Inula britannica* var. *sublanata* | 6 | 7 | 6 | Yes |
| *Karelinia caspia* | 0 | 0 | 1 | Yes |
| *Bidens cernua* | 0 | 1 | 0 | No |
| *Bidens maximowicziana* | 0 | 1 | 0 | No |
| *Bidens tripartita* var. *tripartita* | 0 | 0 | 1 | No |
| *Xanthium strumarium* | 3 | 6 | 6 | No |
| *Eryngium planum* | 2 | 7 | 5 | No |
| *Sium medium* | 0 | 1 | 0 | No |
| *Carum carvi* | 4 | 0 | 0 | No |
| *Vicatia atrosanguinea* | 3 | 0 | 0 | No |
| *Cenolophium denudatum* | 4 | 5 | 4 | No |
| *Heracleum dissectum* | 2 | 0 | 0 | No |

*Note*: Occurrence frequency represents the number of sampling sites (out of 24) in which each species was recorded within each riparian section. Each site consisted of four plots, and species presence was aggregated at the site level.

**Supplementary Table S2** Woody plant species occurrence across river segments

| River section | Species | Life form | Number of sites | Specific site ID |
| --- | --- | --- | --- | --- |
| MOF | *Betula pendula* | tree | 3 | Q1, Q2, Q3 |
| MOF | *Populus laurifolia* | tree | 6 | Q1, Q2, Q4, Q5, Q8, Q9 |
| MOF | *Larix sibirica* | tree | 1 | Q1 |
| MOF | *Rosa acicularis* | shrub | 3 | Q1, Q5, Q8 |
| MOF | *Rosa laxa* var. *laxa* | shrub | 3 | Q3, Q7, Q8 |
| MOF | *Salix turanica* | shrub | 4 | Q6, Q7, Q8, Q9 |
| MOF | *Salix caspica* | shrub | 3 | Q6, Q7, Q9 |
| MOF | *Caragana leucophloea* | shrub | 2 | Q5, Q8 |
| MOF | *Caragana halodendron* | shrub | 1 | Q8 |
| MFF | *Populus laurifolia* | tree | 7 | W1, W2, W3, W4, W5, W6, W7 |
| MFF | *Salix alba* | tree | 5 | W1, W2, W4, W5, W7 |
| MFF | *Elaeagnus oxycarpa* | tree | 3 | W3, W4, W5 |
| MFF | *Salix turanica* | shrub | 5 | W2, W3, W4, W5, W6 |
| MFF | *Tamarix laxa* var. *laxa* | shrub | 3 | W3, W4, W6 |
| MFF | *Caragana leucophloea* | shrub | 1 | W1 |
| MFF | *Caragana halodendron* | shrub | 3 | W1, W4, W5 |
| MFF | *Rosa laxa* var. *laxa* | shrub | 5 | W1, W3, W4, W5, W6 |
| MFF | *Salix caspica* | shrub | 3 | W2, W3, W5 |
| PLF | *Populus laurifolia* | tree | 3 | W8, W11, W12, W13, W14 |
| PLF | *Salix alba* | tree | 6 | W8, W9, W10, W11, W13, W14 |
| PLF | *Elaeagnus oxycarpa* | tree | 7 | W8, W9, W10, W11, W12, W13, W15 |
| PLF | *Populus alba* var. *alba* | tree | 1 | W12 |
| PLF | *Populus nigra* var. *nigra* | tree | 2 | W9, W10 |
| PLF | *Tamarix laxa* var. *laxa* | shrub | 6 | W8, W9, W11, W13, W14, W15 |
| PLF | *Caragana halodendron* | shrub | 3 | W8, W9, W15 |
| PLF | *Rosa laxa* var. *laxa* | shrub | 4 | W8, W11, W12, W13 |
| PLF | *Salix turanica* | shrub | 4 | W8, W9, W11, W13 |
| PLF | *Nitraria sibirica* | shrub | 4 | W8, W10, W11, W13 |

*Note*: The table lists tree and shrub species recorded in at least one site. Numbers in parentheses indicate the total number of sites per river segment ( MOF: n = 9, MFF: n = 7, PLF: n = 8 ). Specific site IDs are provided for transparency.

**Supplementary Table S3**. Data types used for each analysis.

| Analysis | Data type used |
| --- | --- |
| NMDS/PERMANOVA/PERMDISP | Species cover (%) |
| Alpha diversity (HSR) | Number of species per plot |
| Alpha diversity (HSN) | Relative cover of each species (%) |
| Halophyte richness (HaloSR) | Count of halophyte species per plot |
| GLM | Standardized environmental variables (see Table 1) |
| Random forest | Original environmental variables (see Table 1) |

**Supplementary Table S4** Classification of soil pH, total phosphorus (TP), and total nitrogen (TN) levels for assessing stress intensity

| Content rank | 1 | 2 | 3 | 4 | 5 | 6 |
| --- | --- | --- | --- | --- | --- | --- |
| Description | extremely abundant | abundant | moderate | moderately low | deficient | extremely deficient |
| pH | >8.5 | 7.5~8.5 | 6.5~7.5 | 5.5~6.5 | 4.5~5.5 | <4.5 |
| TP ( g·kg^−1^ ) | >2.0 | 1.5~2.0 | 1.1~1.5 | 0.75~1.0 | 0.5~0.75 | <0.5 |
| TN ( g·kg^−1^ ) | >2.0 | 1.5~2.0 | 1.1~1.5 | 0.75~1.0 | 0.5~0.75 | <0.5 |

*Note*: Adapted from Li, Y., Zhao, H., Liu, J. et al. (2024). A framework for selecting and assessing soil quality indicators for sustainable soil management in waste dumps. Scientific Reports, 14, 8491. <https://doi.org/10.1038/s41598-024-58930-x>
